# Supplementary material for: A Transcriptome-Wide Analysis of Psoriasis: Identifying the Potential Causal Genes and Drug Candidates
Source: Int J Mol Sci. 2023 Jul 20;24(14):11717. doi: 10.3390/ijms241411717 (PMC10380797; doi:10.3390/ijms241411717)
Supplement: Supplementary file 1 [file ijms-24-11717-s001.zip › ijms-2454258-supplementary.pdf]

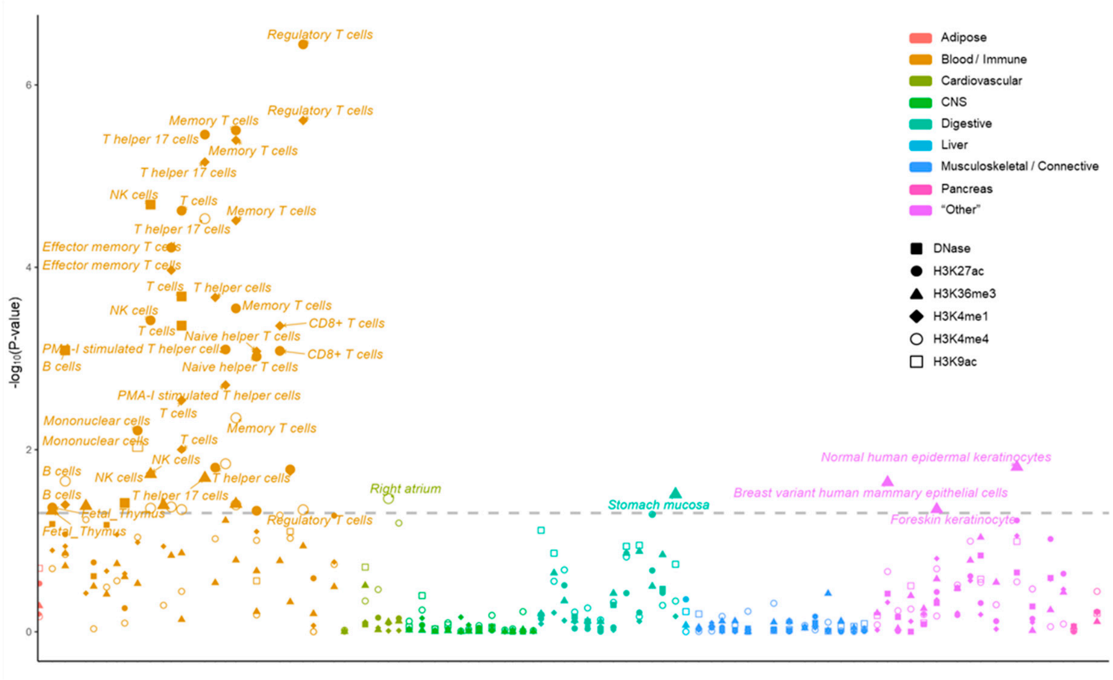

Figure S1. A scatter plot depicting the tissue prioritization via the LDSC-SEG. GTEx data and Franke group's data were divided into nine big categories (scarlet: adipose; mustard: blood/immune; yellow green: cardiovascular; green: central nervous system; turquoise: liver; light blue: musculoskeletal/connective; pink: pancreas; and lilac: "other"). The *Y*-axis denotes -log(*P*-value) and the gray dotted line indicates cutoff (FDR < 0.05).

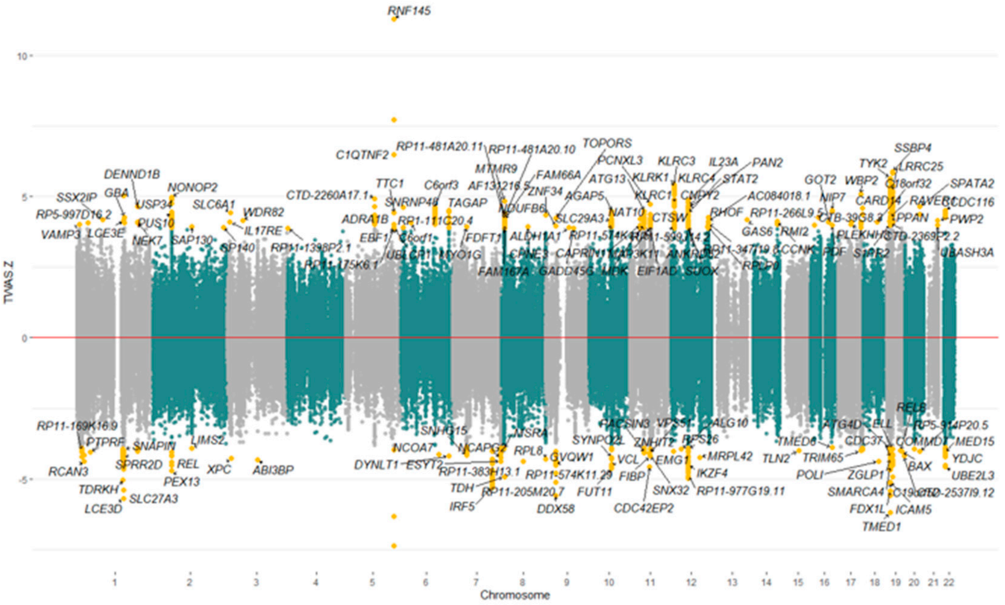

Figure S2. A Manhattan plot showing the result of TWAS in CONTENT panels. The *X*-axis indicates the chromosome number where the gene is located and the *Y*-axis denotes the TWAS-Z-score of

the TWAS signals. Genes that passed the cutoff (FDR < 0.05) were highlighted in yellow. If a gene was identified in several tissues simultaneously, the name of the gene was marked on the largest absolute value.

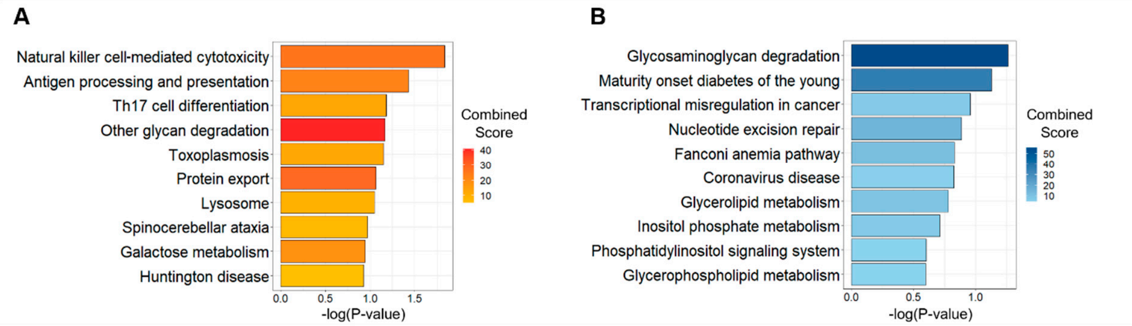

Figure S3. A bar plot depicting the tip 10 enrichment of (A) up-regulated and (B) down-regulated genes in KEGG. The X-axes denote -log(*P*-value) and the color of the bars shows the combined scores from the Enrichr.

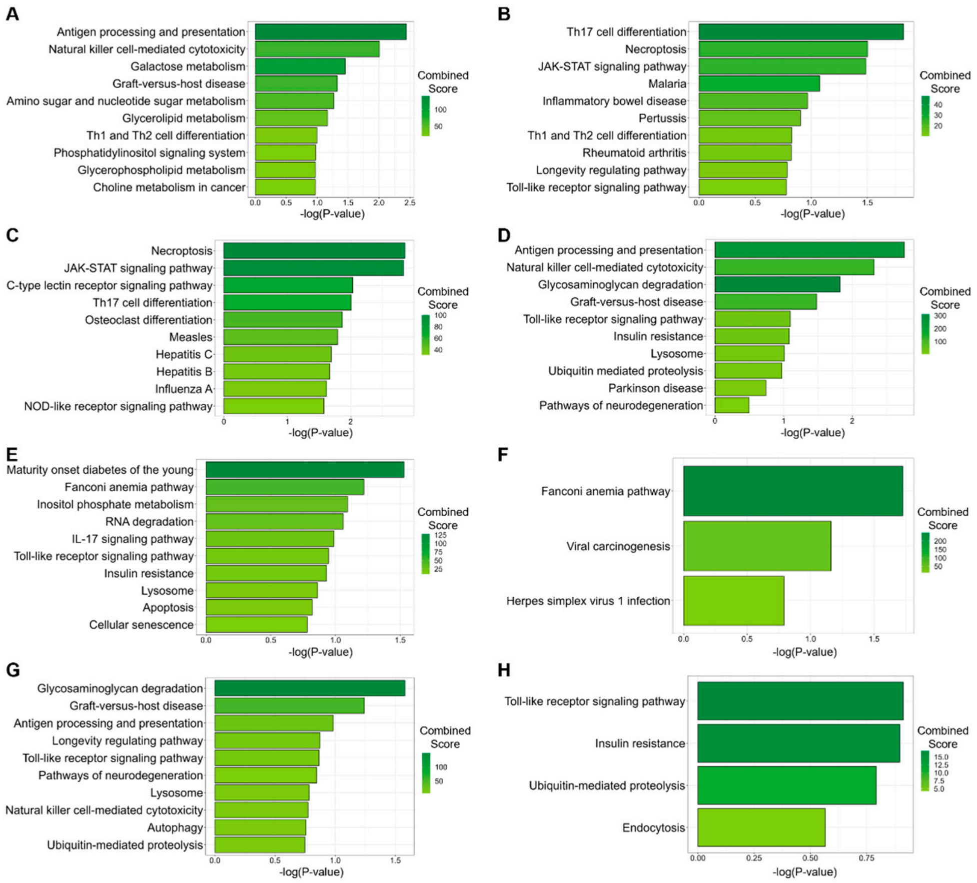

Figure S4. A bar plot depicting the top 10 enrichment of tissue-specific genes in KEGG (A) whole blood, (B) sun-exposed skin, (C) not-sun-exposed skin, (D) spleen, (E) fibroblasts, (F) EBV-transformed

lymphocytes, (G) esophagus mucosa, and (H) stomach. The color of the bars shows the combined scores from the Enrichr.

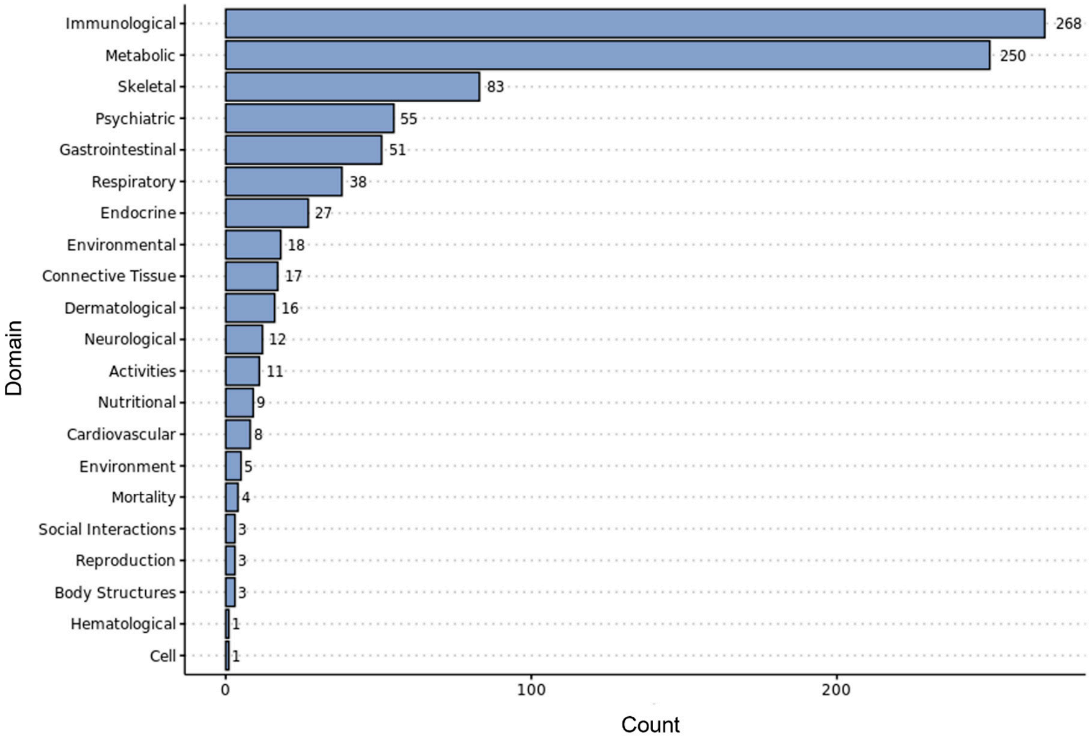

Figure S5. A bar plot of PheWAS using 56 variants from 133 significant psoriasis genes. The *Y*-axis indicates the domains that each phenotype belongs and the *X*-axis indicates the count of the domains. The number located to the right of the blue bar indicates the exact number of each domain.

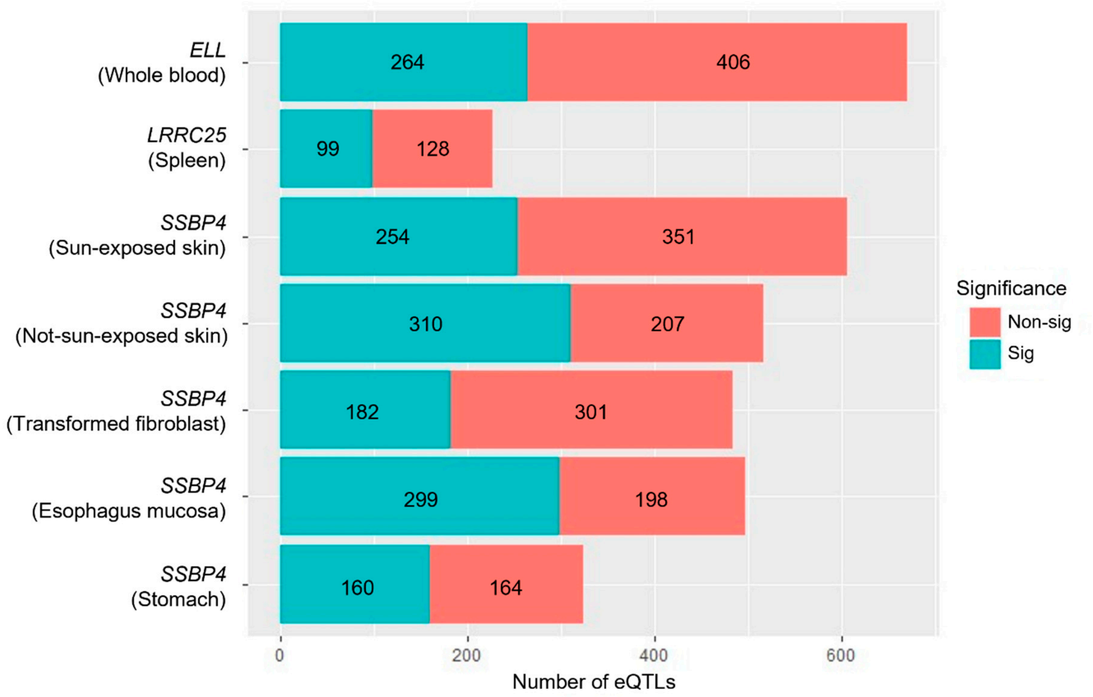

Figure S6. The number of eQTLs of the three novel genes in each tissue. A bar plot showing the number of eQTLs of the three novel genes in each tissue. The *X*-axis denotes the number of total eQTLs and the *Y*-axis denotes tissue-specific three novel genes. The color of the bars indicates the significance of the eQTLs in regulating the gene expression and the number on the bar is the exact eQTL counts of each color.

Table S1. 26 robust genetic markers identified in all the three methods.

Table S2. Results of TWAS using CLUE single-cell panels.

Table S3. Functional annotation results using KEGG pathway.

Table S4. Results of eQTL analysis using GTEx portal. Table S5: Tissue-specific expression data of three novel genes in HPA dataset.
